# Supplementary material for: Indirect punishment can outperform direct punishment in promoting cooperation in structured populations
Source: PLoS Comput Biol. 2025 Jun 2;21(6):e1013068. doi: 10.1371/journal.pcbi.1013068 (PMC12129162; doi:10.1371/journal.pcbi.1013068)
Supplement: S1 Appendix — This file contains two figures, each showing the fractions of the CN, DN, CP, and DP strategies as functions of the punishment cost γ and fine β, under both direct punishment (top row) and indirect punishment (bottom row) scenarios. Figs A and B present results for low dilemma strength (r = 0.05) and high dilemma strength (r = 0.2), respectively (PDF) [file pcbi.1013068.s001.pdf]

Indirect punishment can outperform direct punishment in promoting cooperation in structured populations: S1 Appendix

Yujia Wen, Zhixue He, Chen Shen*, Jun Tanimoto

1 The stable proportion of strategies under direct punishment and indirect punishment

Fig A shows the stable fractions of each strategy under direct and indirect punishment at low dilemma strength ($r = 0.05$), across various combinations of punishment cost (γ) and fine (β) parameters. Throughout the entire cost range ($0 < \gamma < 1$), compared to the baseline case without punishment effects ($\beta = 0$), direct punishment promotes cooperation ($CN + CP$) once the fine β exceeds a certain threshold, which increases with higher γ, as shown in the top panels of Fig A. In contrast, indirect punishment fosters cooperation only within a narrow cost range ($0 < \gamma < 0.12$), where cooperation improves as β increases. Within this range, both direct and indirect punishment can support a fully cooperative state once β surpasses a threshold, indicating their comparable effectiveness in maintaining cooperation. However, beyond this range, changes in β have little impact on the fractions of strategy under indirect punishment. In contrast, as long as β does not fall below γ by a certain margin, direct punishment can sustain a high level of cooperation, demonstrating its superior effectiveness than indirect punishment. When this condition is not met, neither punishment type significantly enhances cooperation, leading to similar outcomes.

At high dilemma strength, the differences between direct and indirect punishment in promoting cooperation align with those observed at low dilemma strength, except when β is lower than γ, where neither type of punishment can sustain cooperation, as shown in Fig B. These results highlight that direct and indirect punishment exhibit distinct thresholds and sensitivities to parameter variations, which leads to divergent effects on strategy distributions across different parameter regimes.

*steven_shen91@hotmail.com

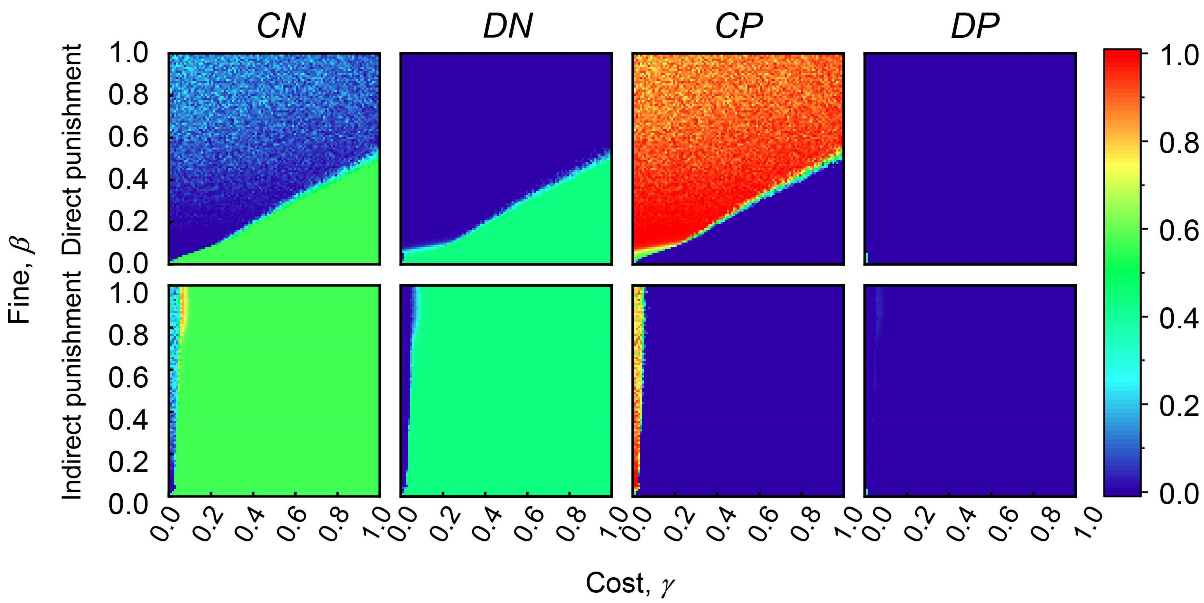

Fig A: Fraction of CN, DN, CP and DP strategy as functions of punishment cost γ and fine β under low dilemma strength $r = 0.05$. The top panels show the results for direct punishment, while the bottom panels display the results for indirect punishment.

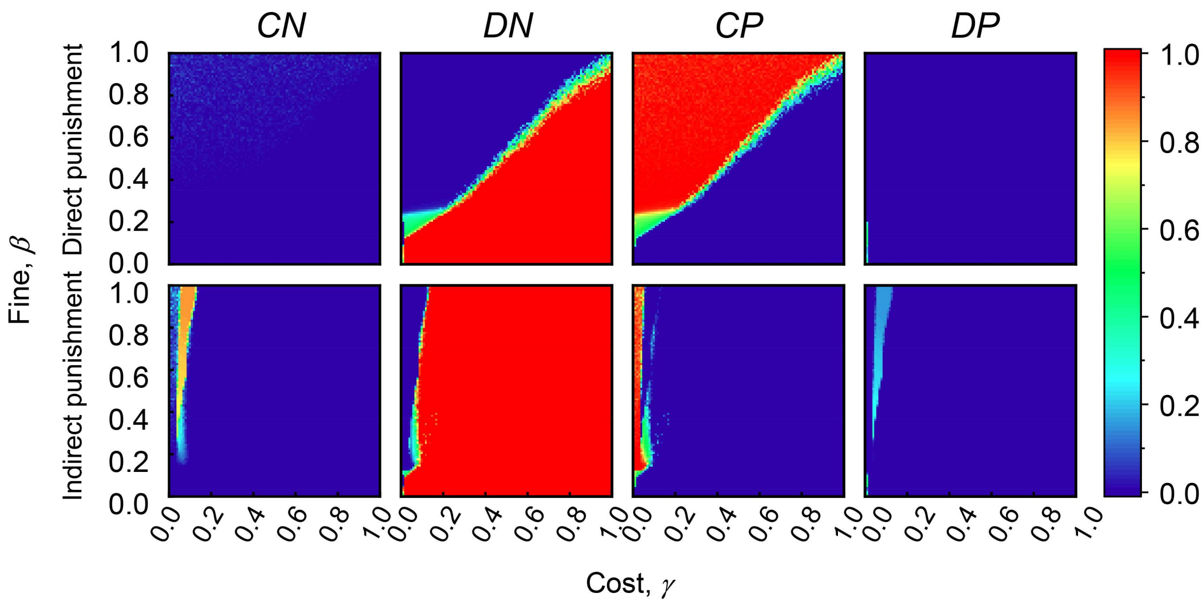

Fig B: Fraction of CN, DN, CP and DP strategy as functions of punishment cost γ and fine β under high dilemma strength $r = 0.2$. The top panels show the results for direct punishment, while the bottom panels display the results for indirect punishment.
